# The Role of Insurance Mechanisms in Managing Zoonotic Risk Within the One Health Approach

**DOI:** 10.3390/healthcare14132022

**Published:** 2026-07-07

**Authors:** Ivanka Lazarova, Desislava Kehayova

**Affiliations:** 1 Faculty of Veterinary Medicine, Trakia University, 6000 Stara Zagora, Bulgaria; 2 Department of Intensive Care and Resuscitation, University Multiprofile Hospital Burgas, 8000 Burgas, Bulgaria

**Keywords:** financial protection, healthcare policy, integrated healthcare systems, health insurance

## Abstract

**Highlights:**

**What are the main findings?**

- Zoonotic diseases create significant health, economic, and social consequences that are intensified by fragmented human, animal, and public health systems.
- Insurance mechanisms may support financial protection, prevention, and early detection, and coordinated management of zoonotic diseases within the One Health framework.

**What are the implications of the main findings?**

- Better coordination between human, animal, and public health systems is necessary for more effective zoonotic risk management.
- Integrated insurance and risk-sharing models may improve prevention, surveillance, resilience and long-term sustainability of One Health systems.

**Abstract:**

Zoonotic diseases represent an increasing health, economic, and social risk in the context of intensified movement of people, animals, and goods, which creates conditions for their rapid spread and the emergence of pandemics. The One Health approach provides an integrated framework that brings together human, animal, and environmental health in order to enable more effective risk management. This study aims to examine the role of insurance and financial mechanisms in zoonotic risk management in the context of One Health. The review identifies four main categories of mechanisms relevant to zoonotic risk management: human health insurance, animal insurance and compensation schemes for livestock producers, public emergency financing mechanisms, and integrated risk-sharing models. The analysis shows that these mechanisms often operate within separate sectors, which may limit their effectiveness in managing complex health threats. These findings suggest that the effectiveness of zoonotic risk management depends not only on the availability of financial resources, but also on how these mechanisms are integrated across sectors. In this context, insurance mechanisms are considered tools for both cost compensation and risk management. They support prevention, early detection, and disease control. The need for their integration within the One Health framework is emphasized. It is also necessary to develop models that ensure a more equitable distribution of risk and financial sustainability for the population as a whole.

## 1. Introduction

Insurance mechanisms in the management of health systems related to human and animal health are increasingly being considered in the context of an integrated approach such as One Health, where the interaction between different subsystems requires coordinated measures and financial sustainability. An integrated approach implies a single platform for the exchange of information regarding human and animal health, including organizational and technical systems such as laboratory centers, sample referral systems, training and human resource development [1,2]. Success depends on the commitment of management at various levels to ensure resources, safety and efficiency through clear procedures and quality control.

The need for integration of human, animal and environmental health is clearly evident in the context of zoonotic diseases. Several zoonotic infections, including tularemia, brucellosis, Q fever, avian influenza, Ebola virus disease, and Nipah virus infection, continue to present challenges for surveillance, prevention and control. Recent outbreaks, together with the COVID-19 pandemic and the growing threat of antimicrobial resistance, have further demonstrated the substantial health, economic, and societal consequences associated with zoonotic diseases and the need for integrated One Health approaches [3,4]. These challenges highlight the importance of coordinated approaches that integrate epidemiological surveillance, veterinary services, public health institutions and sustainable financing mechanisms.

The financial sustainability of health systems is a key element of their functioning. There are a number of examples that show the limitations of classic health financing models, which often operate in a fragmented manner and without sufficient coordination between sectors [5,6]. In addition, division between individual health subsystems can lead to duplication of functions or uneven distribution of resources. This necessitates the development of more flexible mechanisms, including insurance-based ones, to distribute risk and ensure the sustainability of the systems.

There are various financial mechanisms for health risk management described in the literature, including human health insurance systems [5], animal and livestock insurance schemes [7], public compensation funds [8], and risk-sharing mechanisms between the public and private sectors [2].

Socio-economic factors have a significant impact on access to health services and on the effectiveness of insurance systems. Analyses show that the type of health insurance and the level of the premium (the amount of the insurance contribution) correlate with mortality, with groups with a low economic base being particularly vulnerable [9]. This highlights the need for integrated systems to take into account not only medical but also social determinants of health.

Studies on intensive sheep farms have shown high seroprevalence (presence of antibodies in a significant proportion of animals) of *Brucella* spp., *Toxoplasma gondii* and *Coxiella burnetii*, and their spread is associated with risk factors related to the possibility of transmission of the infection, both between the animals themselves and to humans [10]. These infections represent a serious problem both in veterinary practice and in human health risk assessment, and create prerequisites for significant economic losses. This example again clearly demonstrates the need to implement coordinated measures, including financial mechanisms for risk management.

Despite the significant number of studies on zoonotic diseases and the One Health concept, the role of insurance mechanisms as a risk management tool in this context remains insufficiently studied and often considered fragmentarily [11]. Existing research predominantly focuses on surveillance, governance, disease prevention, and intersectoral collaboration, whereas considerably less attention has been paid to the role of health insurance, animal insurance, compensation schemes, and emergency financing mechanisms as integrated components of zoonotic risk management within the One Health framework [12]. In the context of One Health, the integration of epidemiology, health policy and veterinary medicine allows for more effective disease risk management. However, there is a lack of a unified and coordinated approach at the management level that integrates insurance mechanisms between human, animal and public health, which would lead to greater security and an equitable distribution of resources among all stakeholders [12,13]. The experience of the COVID-19 pandemic and other emerging zoonotic threats demonstrates that coordinated actions across human, animal, and environmental health sectors can improve preparedness, strengthen system resilience, and reduce the impact of disease outbreaks [4,14]. In this sense, insurance mechanisms emerge as an essential risk management tool that can support the prevention, early detection and control of zoonotic diseases and contribute to building more resilient health systems.

The aim of this study is to analyze the role of insurance mechanisms in the management of zoonotic diseases within the One Health framework, with particular emphasis on their contribution to different stages of risk management, including prevention, early detection, response, and mitigation of consequences. Special attention is given to the potential of insurance mechanisms to support coordination, risk sharing, and resilience across human, animal, and public health systems. The analysis is structured around the main categories of financial and insurance mechanisms identified in the literature, as well as the opportunities for their integration into risk management models within the One Health framework.

## 2. Materials and Methods

This study was designed as a structured narrative review with a conceptual and policy-oriented synthesis. The aim was to analyse insurance and risk-financing mechanisms relevant to the prevention, preparedness, response, compensation, and recovery phases of zoonotic disease management within the One Health framework.

The literature search was conducted between January and May 2026 using Web of Science, Scopus, PubMed, and Google Scholar. Additional evidence was obtained from reports, technical documents, and policy publications issued by international organisations, including the World Health Organization (WHO), the World Organisation for Animal Health (WOAH), the Food and Agriculture Organization of the United Nations (FAO), the Organisation for Economic Co-operation and Development (OECD), the World Bank, and European Union institutions. Particular emphasis was placed on authoritative international reports and previous review papers, given their relevance for evaluating policy frameworks, financing mechanisms, and cross-sectoral One Health approaches.

The search strategy combined terms related to One Health, zoonotic diseases, insurance, and risk financing, including “One Health”, “zoonotic diseases”, “health insurance”, “livestock insurance”, “risk sharing”, “compensation schemes”, “pandemic preparedness”, “emergency funds”, “public–private partnerships”, and “financial resilience”. Boolean operators AND and OR were used to refine the search results.

Publications and documents published in English between 2003 and 2025 were included if they addressed insurance, compensation, or financing mechanisms relevant to zoonotic disease prevention, preparedness, response, or recovery. Sources unrelated to One Health, zoonotic diseases, insurance, or risk financing, as well as conference abstracts without full text, editorials without analytical content, and duplicate records, were excluded.

**Figure 1:**
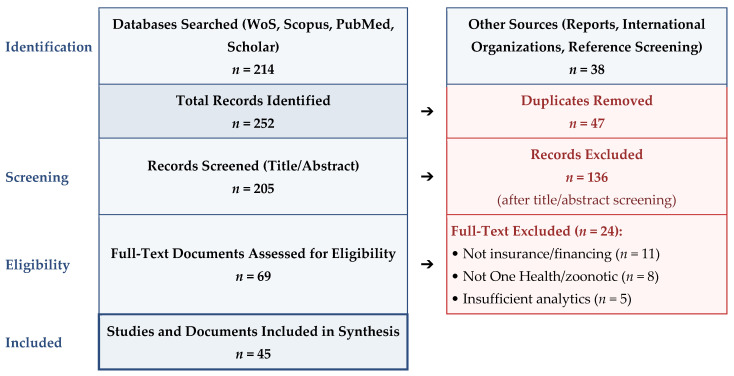
PRISMA 2020 flow diagram of the literature search and screening process.

Titles and abstracts were screened for relevance, followed by full-text assessment of potentially eligible sources. Data were extracted on the type of insurance or financing mechanism, sectoral focus, geographical context, implementation level, reported benefits, limitations, and relevance to One Health risk management. The literature identification, screening, eligibility assessment, and inclusion process is presented in Figure 1.

Generative artificial intelligence tools were used to assist with language editing, improvement of text clarity and structure, and preparation of summary tables. Literature selection, scientific interpretation, data analysis, conclusions, and final manuscript revisions were all performed and critically reviewed by the authors, who take full responsibility for the content of the manuscript.

## 3. Results

### 3.1. Classification of Insurance Mechanisms Relevant to One Health

The literature review indicates that insurance mechanisms relevant to zoonotic risk management can be grouped into four main categories according to their objectives, beneficiaries, and sources of financing. Although these instruments operate across different sectors, all have potential relevance for reducing the health and economic consequences of zoonotic diseases. The identified categories encompass both individual-level financial protection and mechanisms for risk management at the livestock, public health, and intersectoral coordination levels. The proposed classification provides a conceptual framework for the subsequent analysis of their role in zoonotic risk management within the One Health context (Table 1).

The identified mechanisms differ substantially in their target populations, financing structures, governance arrangements and expected outcomes. Human health insurance primarily aims to reduce the financial burden of healthcare costs, whereas animal insurance focuses on compensating livestock losses and supporting disease reporting. Public emergency funds provide resources for outbreak response, while integrated risk-sharing mechanisms seek to strengthen coordination across human, animal and environmental health sectors. However, the reviewed evidence also indicates that no single mechanism is sufficient to address all dimensions of zoonotic risk management, highlighting the need for complementary and context-specific financing approaches.

### 3.2. Characteristics of Zoonotic Risks and Their Economic Impact

Analysis of the reviewed literature indicates that zoonotic risks possess several characteristics that distinguish them from many other health threats. Their transmission across human, animal and environmental systems, combined with uncertainty, potential for rapid spread and significant economic consequences, creates challenges for both risk assessment and resource allocation. The main characteristics of zoonotic risks and their associated economic implications identified in the reviewed literature are summarized in Table 2.

These findings are consistent with previous reports emphasizing the role of surveillance limitations, endemic circulation, environmental drivers and pandemic potential in shaping zoonotic risk [3,26,27].

### 3.3. Human Health Insurance and Individual Financial Protection

People’s health is influenced not only by the availability of medical care but also by access to healthcare services and financial protection against medical expenditures. Evidence indicates that out-of-pocket payments may represent a substantial burden for households and can expose patients to catastrophic health spending. Health insurance mechanisms are designed to reduce this financial risk through different approaches to coverage, financing, and risk sharing. The main human health insurance mechanisms identified in the reviewed literature, together with their advantages and limitations, are presented in Table 3.

### 3.4. Animal Insurance and Livestock Compensation Mechanisms

Unlike human health insurance, which is primarily designed to cover healthcare expenditures, animal insurance and livestock compensation mechanisms are mainly intended to address economic losses associated with animal diseases, mortality, compulsory culling, and production disruptions. The reviewed literature identifies several mechanisms aimed at compensating direct and indirect losses in livestock production and supporting the financial stability of farms during disease outbreaks. The main mechanisms identified in the literature, together with their advantages and limitations, are presented in Table 4.

Examples of livestock insurance include index-based livestock insurance programmes implemented in Kenya and Ethiopia, which have been used to improve the resilience of pastoral communities exposed to livestock losses and livelihood shocks.

### 3.5. Public Emergency Funds and Pandemic Risk Financing

Large-scale outbreaks and pandemics may exceed the capacity of traditional insurance mechanisms and require the mobilization of substantial public resources. The reviewed literature identifies a range of public emergency financing and compensation mechanisms designed to support outbreak response, surveillance activities, implementation of disease control measures, and compensation of affected groups during health emergencies. These mechanisms differ in their sources of financing, beneficiaries, and scope of coverage, but share the common objective of providing financial resources during periods of increased health risk. The main mechanisms identified in the literature are presented in Table 5.

## 4. Discussion

### 4.1. Insurance Mechanisms and the Management of Zoonotic Risks

The reviewed evidence was heterogeneous and included observational studies, policy analyses, international reports, and review papers. Although most studies reported positive effects of insurance and compensation mechanisms on financial protection, disease reporting, and access to services, the magnitude of these effects varied across settings and population groups. Differences in institutional capacity, financing arrangements, and socio-economic conditions suggest that the effectiveness of these mechanisms remains context-dependent. In the context of public health, insurance mechanisms can be seen as a risk management tool, not just a means of covering single costs. Through an integrated and centralized approach, as well as the distribution of financial risk among larger groups of the population, they reduce the individual burden of health costs and create the conditions for more resilient health systems [5,17]. At the same time, the identified insurance and financing mechanisms differ substantially in their objectives, beneficiaries, and scope of application. This suggests that no single mechanism is capable of addressing all dimensions of zoonotic risk on its own, and that effective risk management requires the combination of multiple instruments within a broader risk management framework based on the principles of One Health [2,6].

The results indicate that human health insurance mechanisms are primarily aimed at reducing the financial consequences of disease and improving access to healthcare services. However, even when health insurance coverage is available, gaps in economic protection persist for certain population groups, and full financial protection is not always achieved. For example, Ma et al. [35] found that patients with cardiovascular diseases continued to experience limitations in economic protection, particularly among vulnerable groups with high healthcare needs, while fragmentation between different insurance schemes resulted in unequal protection against financial risk. Although cardiovascular diseases are not associated with zoonotic transmission or epidemic spread, these findings demonstrate that financial difficulties may persist even within well-developed health insurance systems. This suggests that the role of health insurance within the One Health framework should not be limited solely to covering treatment costs, but should also include ensuring equitable access to healthcare services, which is essential for the timely detection, diagnosis, and control of infectious and zoonotic diseases. In livestock production, compensation mechanisms perform not only an economic but also an important epidemiological function. One of the factors that have had the most positive impact on mechanisms for the notification of animal diseases and zoonoses is economic compensation to producers following the implementation of stamping-out measures [20,36].

Delayed disease reporting and the potential for rapid spread of zoonotic diseases necessitate additional financing through public emergency and compensation funds. Sustainable financing and intersectoral collaboration are considered important prerequisites for strengthening surveillance, preparedness, and response capacities, thereby supporting the effective prevention and control of zoonotic diseases [37].

### 4.2. Equity and Socio-Economic Challenges of Insurance Mechanisms

The results of this review indicate that insurance mechanisms can reduce financial vulnerability, but their effectiveness depends largely on their design. Evidence from China shows that the integration of urban and rural health insurance systems reduced the risk of poverty among rural residents by 6.32%, with fully integrated schemes proving more effective than transitional models with multiple insurance arrangements. The study identified increased labour participation and greater use of preventive health services as key mechanisms underlying this effect. These findings suggest that integrated insurance systems not only provide financial protection but also improve access to preventive healthcare, thereby contributing to the development of human capital and population productivity [15,16]. Although the study focused on human health, its findings support the view that equitable and integrated access to healthcare services may facilitate earlier disease detection, particularly among vulnerable populations, and promote the timely implementation of measures to limit disease spread.

Kim et al. [9] reported significant differences in mortality according to both insurance type and premium level, indicating that socio-economic status remains an important determinant of health outcomes despite universal health insurance coverage. In the Korean National Health Insurance System, employee-insured individuals and their dependents account for approximately 70% of enrollees, whereas the self-employed insured group, which includes farmers, represents around 27%. The authors also noted that individuals with higher incomes are more likely to hold supplementary private insurance, which may facilitate access to services and treatments not fully covered by public insurance, potentially widening existing inequalities in health protection. These findings highlight the importance of incorporating equity safeguards into One Health financing models to ensure that access to prevention, diagnosis, treatment, and compensation mechanisms is not determined by socio-economic status. This consideration may be particularly important in low- and middle-income countries, where financial constraints, limited institutional capacity, and fragmented health systems can further restrict access to healthcare services, insurance coverage, and zoonotic disease control measures.

### 4.3. Design Principles and Future Directions for One Health Insurance Models

The findings of the present review indicate that the development of effective insurance models within the One Health framework requires the integration of several interconnected components. First, risk-sharing mechanisms across different population groups can reduce financial vulnerability and the risk of poverty resulting from disease [15,16,25,38]. Second, compensation mechanisms in livestock production can support the early reporting of diseases and the implementation of control measures, thereby reducing both economic losses and the risk of zoonotic disease spread [20,39]. In addition, such mechanisms may reduce incentives for inadequate investment in biosecurity and encourage more timely disease reporting [34].

In addition, large-scale health crises have demonstrated the need for supplementary financial preparedness mechanisms, including public emergency funds and dedicated financing instruments for pandemic prevention, preparedness, and response [23,40].

Last but not least, growing evidence indicates that climate change, land-use change, biodiversity loss, and increasing interactions among humans, domestic animals, wildlife, and ecosystems play an important role in the emergence and spread of zoonotic diseases. Consequently, environmental risk management should be considered an integral component of future risk-management and financing models within the One Health framework [26,41]. Therefore, future models should be based on integrated governance mechanisms, data-sharing frameworks, and coordinated surveillance systems that facilitate the early detection of emerging threats and support timely responses to outbreaks across the human, animal, and environmental health sectors [1,42].

Another important aspect is linking financial mechanisms to prevention activities. The findings of this review indicate that compensation and insurance mechanisms can influence stakeholders’ behaviour and their willingness to report diseases and participate in control measures. Therefore, future models should incorporate incentives related to the implementation of biosecurity measures, vaccination programmes, participation in surveillance systems, and the prudent use of antimicrobials [20,41]. In addition, such mechanisms may reduce incentives for inadequate investment in biosecurity and encourage more timely disease reporting [39]. Their effectiveness, however, depends not only on the availability of financial resources but also on trust in institutions and the perceived fairness of compensation and insurance arrangements. Evidence suggests that trust can influence participation in disease notification and surveillance activities, whereas limited confidence in institutions may undermine compliance with control measures and public health reporting systems [43]. Linking compensation schemes and insurance coverage conditions to compliance with such preventive measures could encourage investments in risk reduction before outbreaks occur, shifting the focus from post-event compensation towards proactive risk management and preparedness.

The findings further indicate that different categories of health threats require different financial instruments. While traditional insurance and compensation mechanisms may be appropriate for more predictable zoonotic diseases, large-scale events such as COVID-19 can generate systemic losses that exceed the capacity of conventional insurance systems. This highlights the need for complementary financing mechanisms, including pandemic funds, public reinsurance arrangements, and rapid-disbursement instruments designed to support prevention, preparedness, and response [23,24,31,44]. Therefore, the integration of innovative financial instruments such as parametric insurance, public–private risk-sharing mechanisms, public reinsurance arrangements, pandemic risk funds, and rapid-disbursement mechanisms triggered by predefined indicators could expand the capacity to manage systemic health risks and provide faster access to financial resources during public health emergencies [23,44].

Future research should focus on evaluating how insurance and financing mechanisms can be integrated across the human, animal, and environmental health sectors within the One Health framework. Particular attention should be given to assessing the effectiveness of integrated financing models in strengthening disease surveillance, supporting early reporting, improving preparedness, and reducing the socio-economic impacts of zoonotic outbreaks. Additional studies are needed to examine issues of equity, access, and affordability, particularly in low- and middle-income countries, where financial and institutional constraints may limit implementation. Furthermore, the development of standardized indicators for evaluating the performance and long-term sustainability of One Health financing mechanisms represents an important research priority.

Taken together, these principles outline a conceptual framework for the development of One Health insurance models that combine financial protection, prevention-oriented incentives, equitable access to services and compensation, integrated surveillance and data-sharing systems, environmental risk monitoring, and rapid response mechanisms within a coordinated cross-sectoral governance structure. Such an approach may strengthen preparedness, resilience, and the long-term sustainability of strategies aimed at preventing and managing zoonotic risks.

## 5. Conclusions

Zoonotic diseases pose a significant challenge to modern health systems, and their management requires an integrated approach that integrates human, animal and environmental health. The One Health concept provides a framework for such integration, but its effective implementation depends on the availability of sustainable financial mechanisms.

The results of this review show that insurance and financial mechanisms can contribute to zoonotic risk management not only through financial protection, but also by incentivizing prevention, early disease reporting and participation in control measures.

The evidence analyzed shows that the different mechanisms perform complementary functions, with health insurance improving access to health services, compensation schemes supporting animal disease control and public funds ensuring financial preparedness for large-scale health crises. However, the effectiveness of these mechanisms depends on their design, the level of integration between sectors and their ability to take into account socio-economic inequalities. From a policy perspective, priority should be given to strengthening surveillance-linked financing mechanisms, aligning compensation schemes with biosecurity and disease-reporting requirements, and promoting cross-sectoral financing arrangements that support coordination across the human, animal, and environmental health sectors.

In summary, future One Health models should combine financial protection, prevention incentives, integrated surveillance and sustainable financing mechanisms. Such an approach can increase the preparedness and resilience of zoonotic risk management systems in the face of growing health and environmental challenges.

## Figures and Tables

**Table 1:** Classification of insurance mechanisms relevant to zoonotic risk management within the One Health framework.

Category	Example of Mechanism (Main Beneficiary)	Main Objective and Reported Benefit	Main Limitation	Relevance to One Health
Human health insurance	Integrated rural health insurance, China (rural households) [15,16]	Reduced risk of impoverishment due to illness and improved access to healthcare services	Variation in benefits across income and population groups	Supports early diagnosis and reduces financial barriers to treatment
	National Health Insurance, Republic of Korea (entire population, particularly low-income groups) [9]	Universal coverage and improved access to healthcare services	Mortality inequalities persist between insurance groups	Facilitates access to healthcare services and disease detection
	Universal health coverage financing approaches (general population) [5,17]	Improved financial protection and access to healthcare services	Requires broader health-system reforms beyond financing mechanisms	Strengthens health-system resilience and access to care during zoonotic disease outbreaks
Animal and livestock insurance	Animal disease compensation schemes and livestock insurance programmes (farmers, livestock producers, and agricultural enterprises) [7,8,18,19,20]	Promotes disease reporting, supports biosecurity measures, and reduces economic losses associated with animal disease outbreaks	Risk of moral hazard, affordability constraints, and uneven coverage	Supports surveillance, outbreak control, biosecurity, and resilience of animal production systems
Public emergency funds	Pandemic Emergency Financing Facility (PEF) and pandemic preparedness financing mechanisms (governments and public health authorities) [21,22,23,24]	Rapid mobilization of financial resources and strengthening of preparedness and response capacity	Dependence on predefined financing conditions and sustained political commitment	Supports emergency preparedness, outbreak response, and financial resilience across sectors
Integrated One Health mechanisms	One Health Joint Plan of Action (FAO–UNEP–WHO–WOAH) and cross-sectoral financing initiatives in Peru (multiple stakeholders) [2,12,25]	Improves coordination, information sharing, and resource mobilization across sectors	Limited implementation experience and lack of long-term evaluation	Supports integrated management of zoonotic and environmental risks

**Table 2:** Main characteristics of zoonotic risks and their economic implications.

Characteristic of Zoonotic Risk	Economic Implications
Uncertainty and limited surveillance	Delayed detection and underestimation of losses
Cross-sector transmission between humans, animals and environment	Need for coordinated control measures and resource allocation
Endemic circulation of pathogens	Long-term healthcare and production costs
Epidemic and pandemic potential	Large-scale economic disruption and emergency expenditures
Antimicrobial resistance	Increased treatment costs and productivity losses
Influence of environmental and climatic factors	Increased unpredictability of outbreaks and prevention costs

**Table 3:** Human health insurance mechanisms and their role in zoonotic risk management.

Insurance Mechanism	Insurance-Related Problem	Advantages	Limitations
Mandatory public health insurance [5,17,28]	High out-of-pocket healthcare expenditures and limited access to medical services	Reduces the financial burden associated with diagnosis and treatment; improves access to healthcare services	Coverage and quality may vary between countries; vulnerable populations may still face barriers to access
Voluntary supplementary health insurance [11,15,16]	Insufficient coverage of services under basic health insurance schemes	Provides additional financial protection and access to a wider range of healthcare services	Access is often associated with income level; may contribute to socio-economic inequalities
Risk pooling through health insurance systems [5,28,29]	Individual exposure to catastrophic healthcare costs during disease episodes	Distributes financial risk across larger population groups and reduces individual exposure to catastrophic healthcare expenditures	Fragmented insurance systems may reduce efficiency and create unequal protection between population groups

**Table 4:** Animal Insurance and Compensation Mechanisms and Their Role in Zoonotic Risk Management.

Insurance Mechanism	Problem Addressed	Advantages	Limitations
Livestock insurance, including index-based livestock insurance programmes in Kenya and Ethiopia [7,18,30,31]	Economic losses resulting from animal diseases, mortality, and reduced productivity	Compensates direct financial losses and supports the economic resilience of farms	Limited implementation in some countries; dependent on the financial capacity of farmers and insurance affordability
Compensation schemes for compulsory culling implemented in European Union animal disease control programmes [32,33,34]	Losses resulting from the destruction of animals as part of disease control measures	Supports compliance with disease control measures and reduces the financial impact on livestock owners	The amount and timing of compensation may vary between systems and jurisdictions
Mechanisms incentivizing early disease reporting [32,33]	Delayed disease notification due to concerns about economic losses	Encourage early detection and containment of outbreaks	Effectiveness depends on trust in the system and the availability of adequate compensation mechanisms

**Table 5:** Public emergency funding mechanisms and their role in zoonotic risk management.

Mechanism	Insurance/Financing-Related Problem	Advantages	Limitations
Public emergency funds [21]	Insufficient financial resources during outbreaks and public health emergencies	Enable rapid mobilization of resources for surveillance, outbreak control and emergency response activities	Limited financial capacity and dependence on available public budgets
Government compensation schemes [19]	Economic losses resulting from disease control measures, movement restrictions and emergency interventions	Reduce the financial burden on affected stakeholders and support compliance with control measures	Compensation levels and eligibility criteria may vary between jurisdictions
Pandemic preparedness and response financing mechanism [21,22]	Need for immediate funding to support public health interventions during large-scale outbreaks	Strengthen response capacity and facilitate implementation of disease control measures	Often activated only after outbreaks occur and may not support long-term prevention activities

## Data Availability

No new data were created or analyzed in this study. Data sharing is not applicable to this article.
